# Chemical Logic Gates on Active Colloids

**DOI:** 10.1002/advs.202305695

**Published:** 2024-03-07

**Authors:** Jiang‐Xing Chen, Jia‐Qi Hu, Raymond Kapral

**Affiliations:** ^1^ Department of Physics Hangzhou Normal University Hangzhou 311121 China; ^2^ Chemical Physics Theory Group Department of Chemistry University of Toronto Toronto Ontario M5S 3H6 Canada

**Keywords:** active colloid, capability of computation, continuum theory, enzymatic network, motor‐based logic gates

## Abstract

Recent studies have shown that active colloidal motors using enzymatic reactions for propulsion hold special promise for applications in fields ranging from biology to material science. It will be desirable to have active colloids with capability of computation so that they can act autonomously to sense their surroundings and alter their own dynamics. It is shown how small chemical networks that make use of enzymatic chemical reactions on the colloid surface can be used to construct motor‐based chemical logic gates. The basic features of coupled enzymatic reactions that are responsible for propulsion and underlie the construction and function of chemical gates are described using continuum theory and molecular simulation. Examples are given that show how colloids with specific chemical logic gates, can perform simple sensing tasks. Due to the diverse functions of different enzyme gates, operating alone or in circuits, the work presented here supports the suggestion that synthetic motors using such gates could be designed to operate in an autonomous way in order to complete complicated tasks.

## Graphical abstract

Small chemical networks that make use of enzymatic reactions on the colloid surface—are proposed to construct motor‐based chemical logic gates. The basic features that are responsible for propulsion and underlie the construction and function of gates are described. Examples are given that show how colloids with specific chemical logic gates, can perform simple sensing tasks.

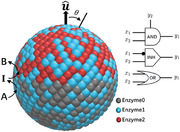

## 1 Introduction

Biological systems utilize complex networks of chemical reactions that produce the chemical species needed to carry out functions such as targeting specific cells in a population of diverse cell types. Likewise, molecular machines powered by chemical energy perform a myriad of specialized tasks, including the transport of cargo to different parts of the cell.^[^ ^1^ ^]^ There is a growing interest in micro and nanoscale synthetic particles that can mimic some aspects of these biological systems and function as vehicles for drug delivery, cargo transport, motion‐based species detection, microfluidics, medical, as well as other applications.^[^ ^2^, ^3^, ^4^, ^5^, ^6^, ^7^, ^8^ ^]^ In this context, extensive investigations of colloidal motors with micrometer dimensions that use various mechanisms to produce propulsion have been carried out, and review articles summarize much of this research.^[^ ^9^, ^10^, ^11^, ^12^, ^13^, ^14^, ^15^ ^]^ Not only can single colloidal motors carry out such tasks, but collections of many active colloids can self‐assemble to form structures that have applications for materials science and other fields.^[^ ^16^, ^17^ ^]^ In order for such micromotors to perform useful tasks effectively, their directed motion must be controlled in some way, even in the presence of strong thermal fluctuations. Chemical gradients, walls, and external fields, among other means, have been used to guide their motions.^[^ ^18^, ^19^, ^20^, ^21^, ^22^, ^23^, ^24^, ^25^, ^26^, ^27^, ^28^ ^]^ Rather than seeking to externally direct motor motion, it would be desirable if the motors themselves could discover ways of responding to stimuli to achieve specific goals.

Chemically‐powered colloidal motors can be propelled using diffusiophoretic mechanisms^[^ ^29^, ^30^, ^31^, ^32^, ^33^, ^34^ ^]^ that make use of non‐symmetric catalytic chemical activity on their surfaces to execute directed motion. Colloidal motors that use enzymatic chemical reactions^[^ ^35^, ^36^, ^37^, ^38^, ^39^, ^40^ ^]^ are the focus of this work. Such enzyme‐powered micro and nanomotors have important properties, such as biocompatibility, versatility, and fuel bioavailability, that make them attractive for applications.^[^ ^39^, ^40^, ^41^, ^42^, ^43^, ^44^ ^]^ Usually the chemical fuel that powers motor motion is supplied directly by the environment; however, active colloids that make use of coupled enzymatic reactions for propulsion and multi‐fueled enzymatic motors have been studied in the laboratory.^[^ ^45^, ^46^, ^47^, ^48^ ^]^ Here we exploit the fact that enzymatic reactions can be constructed to function as logic gates. For example, consider an enzyme that binds two substrates with steep response curves and produces a product species. The product will only be formed if both substrates have sufficiently high concentrations to be bound, so that the enzyme reaction functions as an AND gate, with the substrates being the two inputs and the product the output.

A body of earlier research has shown how DNA, RNA, and protein networks can be used to construct chemical logic gates, and how circuits built from these gates could be used to carry our simple computations.^[^ ^49^, ^50^, ^51^, ^52^, ^53^, ^54^, ^55^, ^56^, ^57^ ^]^ For example, a logic network composed of three enzymes operating in concert as four concatenated logic gates (AND/OR) was designed to process four different chemical input signals and finally produces a pH change as the output signal.^[^ ^58^ ^]^ Also, various two‐input gates built from de novo‐designed proteins have been proposed recently,^[^ ^59^ ^]^ contributing to the design of programmable protein circuits.^[^ ^60^ ^]^

Given the substantial amount of research on the construction of protein and other chemical logic gates and circuits, it should be possible to exploit this research to construct programmable enzyme‐powered motors. Colloids with linear dimensions of one to a few micrometers can support tens to hundreds of thousands of enzymes on their surfaces; thus, one can construct colloids coated with several different enzymes to implement chemical logic gate functions as described above. In this connection, micromotors with a gated pH responsive DNA nanoswitch have been made and studied in the laboratory to function as on‐demand payload delivery systems.^[^ ^42^, ^43^ ^]^

In this paper we investigate various ways in which small chemical networks that make use of enzymatic chemical reactions on the colloid surface can be used to construct motor‐based chemical logic gates. In this way the motors may perform chemical computational tasks that allow them to control their dynamics by sensing the characteristics of the environment in which they move. Below we present a discussion of some basic aspects of how coupled enzyme reactions influence colloid propulsion, and provide examples of how specific chemical logic gates can be implemented to allow a colloidal motor to sense and respond to its environment in different ways, thus, performing simple tasks.

## 2 Results and Discussion

### 2.1 Chemical Gates and Colloidal Motors with Coupled Enzymatic Reactions

When a number of different enzymes are attached to the surface of a colloid with specific spatial distributions, the enzymatic reactions they catalyze can form a small chemical network. Some of these reactions may give rise to colloid propulsion or participate indirectly in the propulsion mechanism, while others may be used for environmental sensing and response. Also, such colloidal enzyme networks can be constructed to act as logic gates.

The majority of chemically‐active colloidal motors that have been studied experimentally are Janus particles with catalytic and non‐catlaytic faces. Often, one face is coated by a metal such as Pt that catalyzes the decomposition of hydrogen peroxide (H_2_O_2_) fuel to effect propulsion. Also, enzyme coated hollow silica colloids with either Janus^[^ ^3^, ^36^, ^37^, ^40^ ^]^ or random inhomogeneous enzyme distributions have been constructed in the laboratory.^[^ ^61^ ^]^

In order to show how small colloidal chemical networks operate, in this section we present simulation and continuum theory modeling of an active Janus colloid with two coupled enzymatic reactions that involve local fuel supply. A laboratory example^[^ ^46^, ^47^, ^48^ ^]^ is provided by a Janus colloid half coated by catalase that uses the catalytic decomposition of H_2_O_2_ to produce propulsion; however, instead of the more conventional global fuel supply, the H_2_O_2_ fuel is supplied locally to catalyze by reactions at a second enzyme, glucose oxidase (GO_*x*_). In this system the reaction of glucose (Glc) in the presence of O_2_ catalysed by GO_*x*_ yields gluconic acid (GlcA) and H_2_O_2_, $\mathrm{Glc}+O_{2}\overset{{\text{GO}}_{x}}{\to }H_{2}O_{2}+\mathrm{GlcA}$. This reaction, in turn, supplies the H_2_O_2_ fuel that is used by catalase in the reaction $H_{2}O_{2}\overset{\text{Cat}}{⇌}H_{2}O+O_{2}$ to propel the colloid. Local supply of fuel will allow the catalyse motor to operate under conditions where the bulk phase concentration of H_2_O_2_ is low, and this could be advantageous in biological applications since H_2_O_2_ is a toxic fuel. We also note that coupled chemical reactions on active flexible sheets have also been studied and can be used to control their dynamics.^[^ ^62^ ^]^

**Figure 1:**
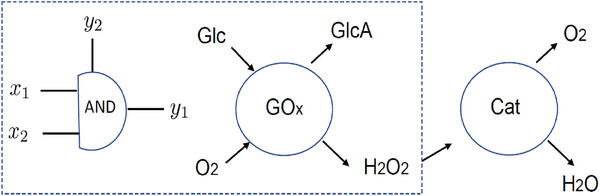
A small protein network that simulates an AND gate whose output is a species that is fuel for a second enzyme. The glucose and oxygen substrates correspond to the inputs *x*_1_ and *x*_2_ to the AND gate, while hydrogen peroxide is the desired output *y*_1_. The product *y*_2_ = GlcA is not monitored. The hydrogen peroxide is then processed by the catalase enzyme to produce O_2_ and H_2_O.

This mechanism also contains the basic elements needed to form an AND gate. Indeed, **Figure** **1** shows how the reaction catalyzed by GO_*x*_ can be mapped onto an AND gate. The inputs to the gate are *x*_1_ = Glc and *x*_2_ = *O*_2_, while the target output is hydrogen peroxide, *y*_1_ = H_2_O_2_. The GlcA product is not monitored. The reaction models an AND gate since both substrates are required for product formation.^[^ ^63^ ^]^

**Figure 2:**
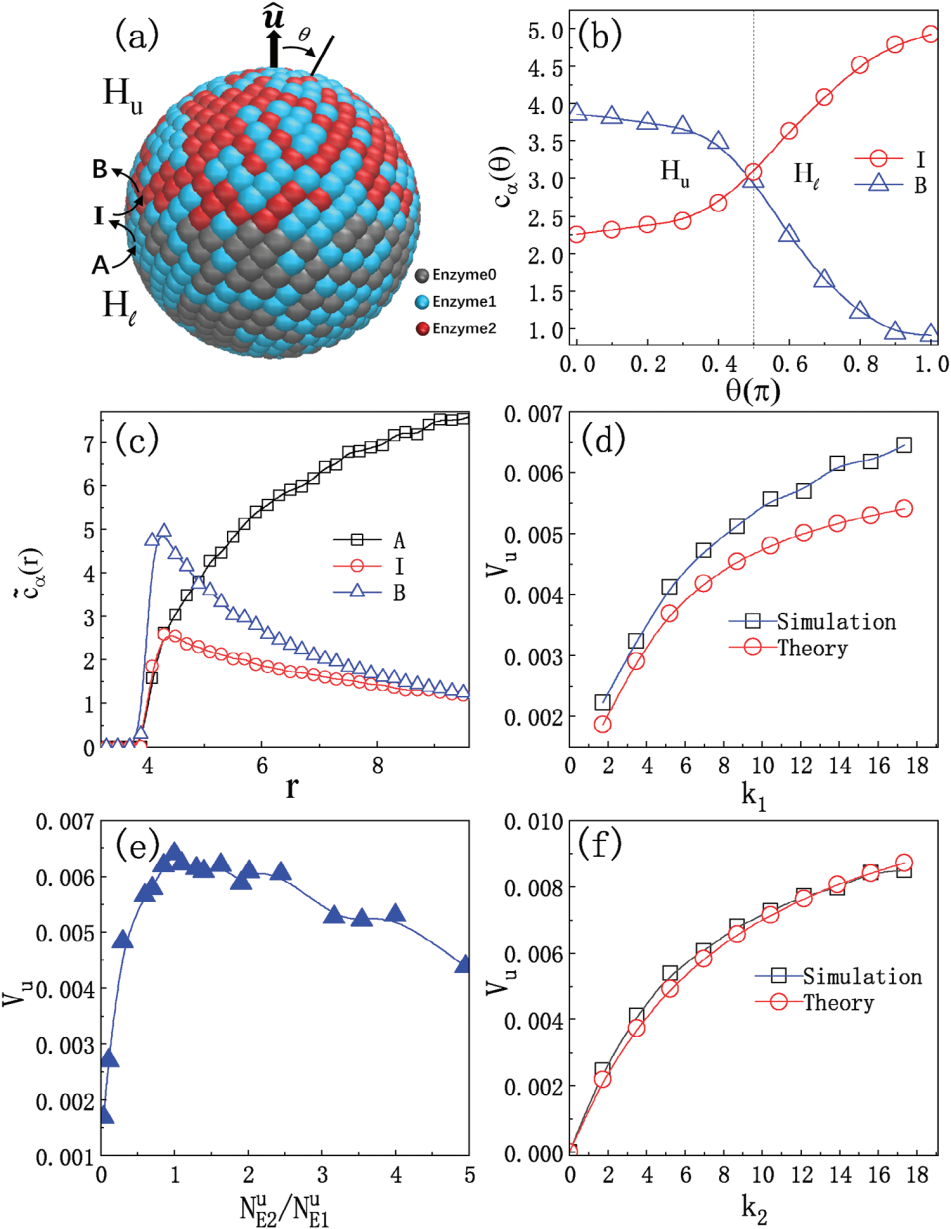
a) A colloidal Janus motor with radius *R* = 4.0 and 1000 coarse‐grained enzymatic sites on its surface. The fractions of E_1_ sites (blue) on the two hemispheres are $f_{1}^{u}=f_{1}^{\ell }=1/2$, so that E_1_ and E_2_ each occupy 250 sites on the *H*_*u*_ cap, while there are 250 E_1_ and 250 E_0_ sites (gray) on the *H*_ℓ_ cap. The motor axis is defined by the unit vector $\hat{u}$ pointing from the center‐of‐mass of the colloid to that of the *H*_*u*_ cap containing E_2_ sites (red). The energy parameters are ϵ_A_ = ϵ_I_ = 1.0 and ϵ_B_ = 0.1. b) The intermediate fuel species I (circles) and product B (triangles) concentrations *c*_α_(θ) versus θ at *r* = 5.0. c) The *H*_*u*_‐cap angle‐averaged radial concentrations ${\tilde{c}}_{\alpha }\left(r\right)$ for three substrate species, A (squares), I (circles), and B (triangles). d) Velocity *V*_*u*_ as a function of the reaction rate constant *k*_1_ for fixed *k*_2_. The squares and circles are calculated from simulation and theory, respectively. e) The dependence of *V*_*u*_ on the ratio $N_{E_{2}}^{u}/N_{E_{1}}^{u}$. f) The velocity *V*_*u*_ from simulation (squares) and theory (circles) as a function of *k*_2_ when fuel I is supplied from the bulk. The data is from averages over ten independent realizations.

A simple version of such coupled chemical reactions on the colloid surface can serve to illustrate the roles of various factors, such as reaction rates, manners of fuel supply, and enzyme distributions, on the colloid dynamics. For a spherical colloid with radius *R*, we suppose that on the upper hemisphere (*H*_*u*_) a fraction $f_{1}^{u}$ of surface area is covered by enzyme E_1_, randomly distributed on that hemisphere, while the remaining fraction $f_{2}^{u}=1-f_{1}^{u}$ is covered by enzyme E_2_ (see sketch in **Figure** **2**a). On the lower hemispherical cap (*H*_ℓ_) a fraction $f_{1}^{\ell }$ is covered by enzyme E_1_, while on the remainder of the hemispherical surface the fraction $f_{0}^{\ell }=1-f_{1}^{\ell }$ sites are inactive enzymes E_0_ or empty sites.

Enzyme 1 catalyzes the reaction

(1) $$A+R_{1}^{*}\underset{E_{1}}{\overset{\kappa_{1}}{\to }}I+P_{1}^{*},\;\mathrm{on}\;\mathrm{enzyme}\;1\;\mathrm{surface}\;\mathrm{fractions}$$

where species with a superscript * are assumed to be held constant by reservoirs or are in excess. The fixed concentration $c_{R_{1}}^{*}$ of $R_{1}^{*}$ is incorporated in the rate constant *k*_1_ and κ_1_ = *k*_1_/(4π*R* ^2^) is the rate constant per unit surface area. (If $c_{R_{1}}^{*}$ is in excess or above a threshold the reaction (1) is controlled by the concentration of A. In this case, one input is always on, and the reaction can act effectively as a buffer gate that produces species I if A is an input.) Enzyme 2 catalyzes the reaction,

(2) $$I\underset{E_{2}}{\overset{\kappa_{2}}{\to }}B,\;\mathrm{on}\;\mathrm{enzyme}\;2\;\mathrm{surface}\;\mathrm{fractions}$$

with κ_2_ = *k*_2_/(4π*R* ^2^). In these reactions we see that the product I produced on the motor surface by reaction (1) serves as the fuel for the motor reaction (2). To maintain the system in a non‐equilibrium state, as in many biological contexts, we suppose that B and I are removed from the system by catabolic reactions and A is supplied by other bulk phase reactions. In simulations we model these processes by the reactions $B\overset{k_{b}^{B}}{\to }A$ and $I\overset{k_{b}^{I}}{\to }A$, that take place in the fluid phase with rate constants $k_{b}^{B}$ and $k_{b}^{I}$.

This scheme could also model reactions catalyzed by other enzymes; for example, the enzyme choline oxidase (CHO) that catalyzes the reaction of choline (Ch) and *O*_2_ to give betaine aldehyde (Be) and H_2_O_2_, $\mathrm{Ch}+O_{2}\overset{\text{ChO}}{\to }H_{2}O_{2}+\mathrm{Be}$.^[^ ^55^ ^]^

Instead of a single catalytic reaction, the reaction network using both Equations (1) and (2) also can model an AND gate using the coupled reactions of two enzymes such as GO_*x*_ and horseradish peroxidase (HRP). The H_2_O_2_ from the GO_*x*_ catalysis is then processed by HRP in the reaction, $H_{2}O_{2}+\mathrm{ABTS}\overset{\text{HRP}}{\to }O_{2}+{\mathrm{ABTS}}_{ox}$. With the O_2_ $(R_{1}^{*}$ above) concentration fixed, one can take as inputs *x*_1_ = Glc and *x*_2_ = ABTS, where ABTS is 2,2'‐azino‐bis(3‐ethylbenzothiazoline‐6‐sulfonic acid) and the output of the AND gate is ABTS_*ox*_, the oxidized form of ABTS.^[^ ^55^ ^]^

#### 2.1.1 Continuum Model

The velocity of the motor can be computed using the formula for the diffusiophoretic propulsion velocity^[^ ^29^, ^30^, ^31^, ^32^, ^34^ ^]^ to give^[^ ^64^ ^]^

(3) $$\begin{array}{c} V_{u}=V_{u}\hat{u}=\frac{k_{B}T}{\eta }\left[\Lambda_{\text{IA}}{\bar{\nabla_{s}c_{I}\left(r\right)}}^{S}+\Lambda_{\text{BA}}{\bar{\nabla_{s}c_{B}\left(r\right)}}^{S}\right] \end{array}$$

where the overline denotes an average over the surface of the colloid, and $\Lambda_{\alpha \alpha^{\prime }}=\int_{R}^{R+r_{c}}dr\;\left(r-R\right)\left(e^{-\beta V_{\alpha c}}-e^{-\beta V_{\alpha^{\prime }c}}\right)$ accounts for the interactions of solute molecules of type α with the colloid through repulsive potentials *V*_α_ with finite range *r*_*c*_. The velocity is directed along the unit vector $\hat{u}$ that points from the *H*_ℓ_ to *H*_*u*_ hemispheres, as shown in Figure 2a. The numerical values of the motor velocity can be found by substituting the solutions of the reaction‐diffusion equations, subject to boundary conditions on the motor surface, into Equation (3). This calculation is given in the Experimental Section. Among other factors, the propulsion velocity depends on the rate constants *k*_1_ and *k*_2_, and the fractional coverage.

By contrast, if the fuel *I* is supplied directly from the fluid phase, as is usually done for diffusiophoretic motors, we have

(4) $$V_{u}=\frac{k_{B}T}{\eta }\Lambda_{\text{IB}}{\bar{\nabla_{s}c_{I}\left(r\right)}}^{S}$$

The full expression is also given in the Experimental Section.

Comparisons of Equations (3) and (4) allow one to quantify the differences between fuel supply at the motor surface and supply from the environment. Not only do two rate constants, *k*_1_ and *k*_2_, appear when fuel is supplied locally, but the bulk phase reactions and boundary conditions couple all three concentrations, *c*_A_, *c*_B_, and *c*_I_. Hence, surface fuel production leads to a rich structure that could be exploited to control motor motion.

#### 2.1.2 Simulation

Simulation of the motor dynamics is based on a coarse‐grained microscopic description of the entire system. The surface of the colloid is covered by spherical beads that act as coarse‐grained groups of enzymes. The E_1_ and E_2_ enzyme groups are uniformly and randomly distributed on the colloid surface with fractions as described above and sketched in Figure 2a. The reactions on the surface of the colloid take place on the chemically active enzymatic sites. The strengths of the repulsive colloid‐solute potentials are gauged by ϵ_α_ energy parameters. The evolution of the system is carried out using hybrid molecular dynamics‐multiparticle collision dynamics^[^ ^65^, ^66^, ^67^, ^68^ ^]^ Further information on the simulation model and parameters are given in Experimental Section.

#### 2.1.3 Concentration Fields and Motor Velocity

From Equation (3) one can see that the concentration gradient fields in the colloid vicinity play an important part in the diffusiophoretic mechanism and, for the sequential reaction mechanism of interest here, the results of simulations and continuum calculations allow one to assess the factors giving rise to the spatial structure of these fields. Specifically, the gradient fields ∂_θ_ *c*_B_(*R*, θ) and ∂_θ_ *c*_I_(*R*, θ) enter the expression for the *V*_*u*_ in Equations (3) and (25). Plots of *c*_B_(5, θ) and *c*_I_(5, θ), obtained from averages over the azimuthal angle, versus θ are shown in Figure 2b for a radius value *r* = 5, somewhat outside the boundary region at *r*_c_ = 4.125 where the potentials act. The *c*_*B*_ and *c*_*I*_ profiles are typical of Janus colloids, where the largest gradient is in the vicinity of the equator where the *H*_*u*_ and *H*_ℓ_ caps meet. One also observes the depletion of locally‐supplied I on the upper hemisphere. The pronounced asymmetry of these fields around the colloid indicates that local fuel can provide effective self‐propulsion.

The overall structure of the concentration fields of all three species, α = A, I, B near the *H*_*u*_ cap is seen in the plots of the radial concentrations, ${\tilde{c}}_{\alpha }\left(r\right)$, in Figure 2c. These radial concentrations were constructed from averages of the species density fields only over the angles corresponding to the *H*_*u*_ cap. The plots show that I is efficiently converted to product B in the motor vicinity, while species A remains in high concentration farther from the colloid. Thus, the colloid is able to function as a motor without high concentrations of the undesirable fuel I in the bulk phase, an important feature for biological applications where undesirable fuels such as H_2_O_2_ are required. Furthermore, the consumption characteristics of the intermediates can have an influence on the design of motor chemical gates based on coupled enzyme networks.

Results for the propulsion velocity of the motor are given in Figure 2d–f. The component of the average motor velocity along $\hat{u}$ may be computed in the simulation from $V_{u}=\left\langle V\left(t\right)\cdot \hat{u}\right\rangle$, where V(t) is the instantaneous velocity of the center‐of‐mass of the colloid and the angular brackets denote an average over time and different realizations of the dynamics. The simulation values are compared to the continuum results using Equations (3) and (25) in Figure 2d for different values of *k*_1_ and fixed *k*_2_. In the simulation, *k*_1_ is varied by changing the reaction probability $p_{1}^{R}$ on E_1_ with $p_{2}^{R}=1.0$ for E_2_. In the continuum model, these rate coefficients are estimated from the kinetic theory expression in the Experimental Section. (The abscissa values in the plots are the kinetic theory rate coefficients.) The increase in *V*_*u*_ with *k*_1_ is expected since more fuel is supplied locally to the E_2_ enzymatic reaction that powers propulsion; however, the deviation from linear increase can be attributed to the inability of E_2_ to process the fuel quickly enough before I escapes to the bulk phase. Both simulation and continuum theory are in qualitative agreement and show this effect. The quantitative discrepancy is likely due to correlations in the microscopic coupled surface reaction kinetics on E_1_ and E_2_ that are not captured by the homogeneous reaction model on the catalytic cap used in the continuum theory.

The velocity *V*_*u*_ also depends on the fractional occupancy of E_1_ and E_2_ on the *H*_*u*_ cap, $f_{2}^{u}/f_{1}^{u}=N_{E2}^{u}/N_{E1}^{u}$ as shown in Figure 2e. The rapid increase of *V*_*u*_ with $N_{E2}^{u}/N_{E1}^{u}$ reflects the corresponding increase in local fuel supply from the increase in the numbers of E_2_ enzymes, while the decrease for larger values of $N_{E2}^{u}/N_{E1}^{u}$ is due to the decrease in the numbers of E_1_ enzymes, leading to a maximum at $N_{E2}^{u}/N_{E1}^{u}\approx 1$. These results indicate that choice of the optimal fractional occupancy of E_1_ and E_2_ gives rise to the most powerful propulsion, a feature that could be exploited when designing motors with coupled enzyme kinetics.

The above results are compared to those where fuel I is directly supplied from the bulk in Figure 2f. The colloid now has a fraction $f_{2}^{u}=1/2$ of E_2_ sites on the *H*_*u*_ cap, while all other sites on the colloid are chemically inactive E_0_ sites. Only the reaction rate *k*_2_ enters this calculation (see reaction (7) and Equation (35) in the Experimental Section). The plots are similar to those in panel (d), although the velocity is somewhat larger. Last, global fuel supply simulation and continuum theory are in quantitative agreement, as shown in Figure 2f. As seen above, for local fuel supply the continuum model is not able to quantitatively capture the full reactive dynamics in the boundary layer since the reaction rates it uses are averages over the entire hemisphere.

### 2.2 Motors with Chemical Gates that Sense Their Environments

Since the active colloids respond to both self‐generated and external chemical gradients,^[^ ^29^, ^30^, ^31^, ^32^, ^33^ ^]^ we can exploit this dependence using chemical logic gates to direct their motion. We now show how other chemical logic gates can be used by motors to sense certain aspects of their environments and respond to these chemical signals by carrying out specific tasks. The examples are simple and intended to be illustrative, although the tasks the colloids perform are not very challenging.

#### 2.2.1 INH Gate

The first task is to prevent a colloid from being captured by a source. We suppose that the system contains a colloid (C) and a fixed spherical source (S) of a chemical species I. The colloid responds to the gradient field of this species (I) through a diffusiophoretic mechanism and may be attracted to the source. We want the motor to sense the magnitude of this concentration field and respond to it in a way that prevents capture by the source. We do this by implementing an inhibitor (INH) gate on the colloid.

**Figure 3:**
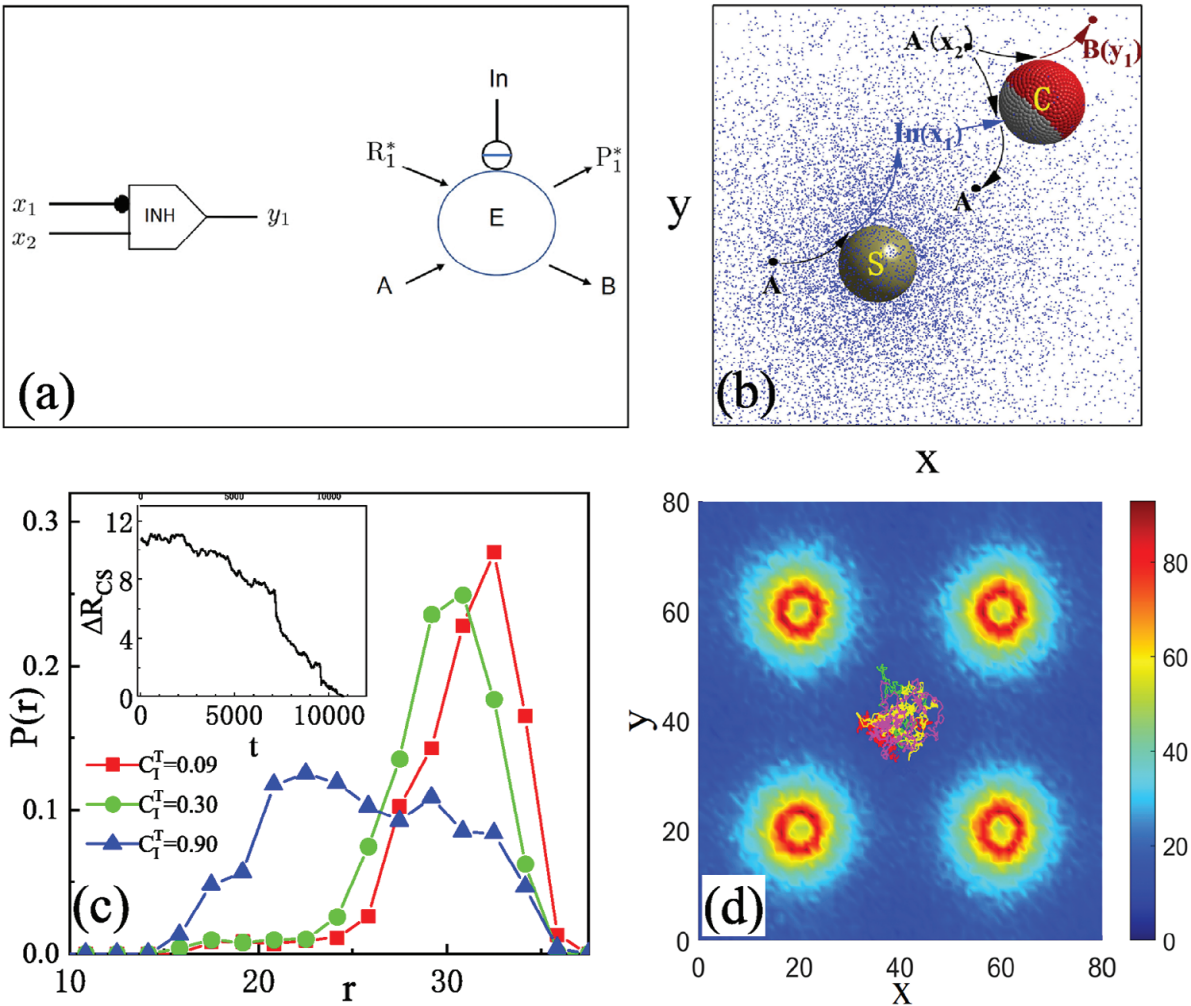
a) An enzymatic reaction that simulates an inhibitor INH gate. The enzyme E catalyzes the reaction of substrates A and $R_{1}^{*}$ to form products B and $P_{1}^{*}$. The open circle with a bar signifies that species In is an inhibitor for the reaction. The substrate A and inhibitor In correspond to the inputs *x*_1_ = In and *x*_2_ = A to the INH gate, while the output is *y*_1_ = B. b) Catalytic source sphere (S, gray) with a radius *R*_s_ = 3.0 and a colloidal sphere (C, red) with radius *R*_c_ = 4.0, along with the *c*_I_ concentration field (blue particles) near the source. The gray beads on the surface of the colloid signal that their enzymatic activities are inhibited by the *I* particles. The repulsive potential parameters are ϵ_A_ = 2.0, ϵ_B_ = 0.2, and ϵ_I_ = 0.1. c) The probability distribution function *P*(*r*) of the distance of the colloid from the source. Three examples with different gate thresholds $c_{I}^{T}=0.09,0.3,0.9$, are plotted. Inset: the evolution of distance Δ*R*_CS_ = ∣**r**_c_ − **r**_s_∣ − (*R*_s_ + *R*_c_) between the source and the colloid when I is not an inhibitor. d) The concentration profile of species I and trajectories of the colloid obtained from five independent realizations of the dynamics in a system with size *L*_*x*_ = *L*_*y*_ = 80 and *L*_*z*_ = 14 containing four source spheres with $c_{I}^{T}=0.3$.

Protein logic gates can be constructed using the properties of inhibitors that prevent certain reactions from taking place. For this purpose, we consider the reaction scheme in **Figure** **3**a which represents an INH gate. This gate is modeled on earlier work for a NOT gate that employs inhibitor molecules.^[^ ^49^ ^]^ In this scheme, an enzyme (E) catalyzes the reaction $R_{1}^{*}+A\overset{E}{\to }P_{1}^{*}+B$, where the * on species $R_{1}^{*}$ and $P_{1}^{*}$ again signifies that they are held fixed by reservoirs. The presence of an inhibitor (In) prevents the reaction from taking place. (The inhibitor gate can also be constructed from NOT and AND gates.) A colloid with an INH gate can sense the concentration of inhibitor in the environment and decide to open or close the activity of the enzyme network on the motor. The colloid responds to the state of the gate by changing its motion.

In the simulations, the colloid and source spheres with radii *R*_c_ and *R*_s_, respectively, are contained in a slab‐shaped volume with dimensions *L*_*x*_ = *L*_*y*_ = 50 and *L*_*z*_ = 14, with two confining parallel walls separated by a distance *L*_*z*_. Periodic boundary conditions are used in the *x* and *y* directions. The initial separation between the S and C spheres is $R_{\text{CS}}^{0}$ = 17.7. The fixed sphere S acts as a source of species I by catalyzing a reaction A → I. The freely‐moving colloid C is uniformly coated by an enzyme E that catalyzes the reaction $A\overset{E}{\to }B$. As in the previous section, there are fluid phase reactions, $I\overset{k_{b}^{I}}{\to }A$ and $B\overset{k_{b}^{B}}{\to }A$, that maintain the system in a non‐equilibrium state. The S sphere produces inhomogeneous A and I concentration fields in its vicinity (see Figure 3b) by

(5) $$c_{I}\left(r\right)\propto \frac{1}{r}e^{-\kappa_{I}\left(r-R_{s}\right)}$$

where $\kappa_{I}=\sqrt{k_{b}^{I}/D}$, with D the solute diffusion coefficient, is the inverse screening length that determines how rapidly the I concentration decays to zero in the bulk. A similar expression can be written for the *c*_A_(*r*) field. The energy parameters are chosen to satisfy ϵ_A_ > ϵ_B_ > ϵ_I_.

Since the catalyst *E* is uniformly distributed over the entire surface of the colloid, in the absence of the source sphere and with a uniform supply of fuel A, the net propulsion force is zero by symmetry. When the S sphere is present and the colloid enters its vicinity, it will experience a depletion of A fuel on its face that points toward the source. The subsequent reaction $A\overset{E}{\to }B$ converts A around the colloid to B. Since ϵ_B_ > ϵ_I_ the diffusiophoretic force will cause the colloid to move toward the S sphere and be captured by it, as shown in the inset in Figure 3c.

To avoid the capture of colloid by the source, an INH gate is constructed on the colloid. Species I acts as the inhibitor (In). If the local concentration of I around an enzyme bead, *c*_I_, exceeds a pre‐set threshold value, $c_{I}^{T}$, we suppose the reaction $A\overset{E}{\to }B$ is inhibited. If the value is either lower or higher than the threshold, the output of the gate is defined to be either 0 or 1, respectively. Therefore, when the colloid moves toward the source, the catalytic activities of enzymes facing the source are suppressed if $c_{I}>c_{I}^{T}$. In this circumstance, the local concentration of *A* is greater than that on the face where the A → B reaction takes place and, since ϵ_A_ > ϵ_B_ > ϵ_I_, a diffusiophoretic force pushes the colloid away from the source. Figure 3c shows the probability density of the colloid distance from the source, *P*(*r*) = 〈δ(∣(**r**_C_ − **r**_S_)∣ − *r*)〉 where **r**_C_ and **r**_S_ are the center‐of‐mass positions of the colloid and source, respectively. The angle bracket denotes an average over time and realizations. When the INH gate is operative, one sees that the colloid cannot be captured by the source. Figure 3c illustrates that for lower thresholds, the colloid tends to remain at large distances from the source with quite sharp probability distributions, while for a large threshold, it is able to explore regions closer to the colloid with a broad distribution, without collapsing to a bound pair.

In Figure 3d we consider a system with a colloid and four source spheres. The gate threshold is set to have an intermediate value, $c_{I}^{T}=0.3$. One sees that the colloid can move among the array of sources without touching them and, in fact, is trapped in a small region between the sources as a result of the action of the INH gate.

#### 2.2.2 OR Gate

**Figure 4:**
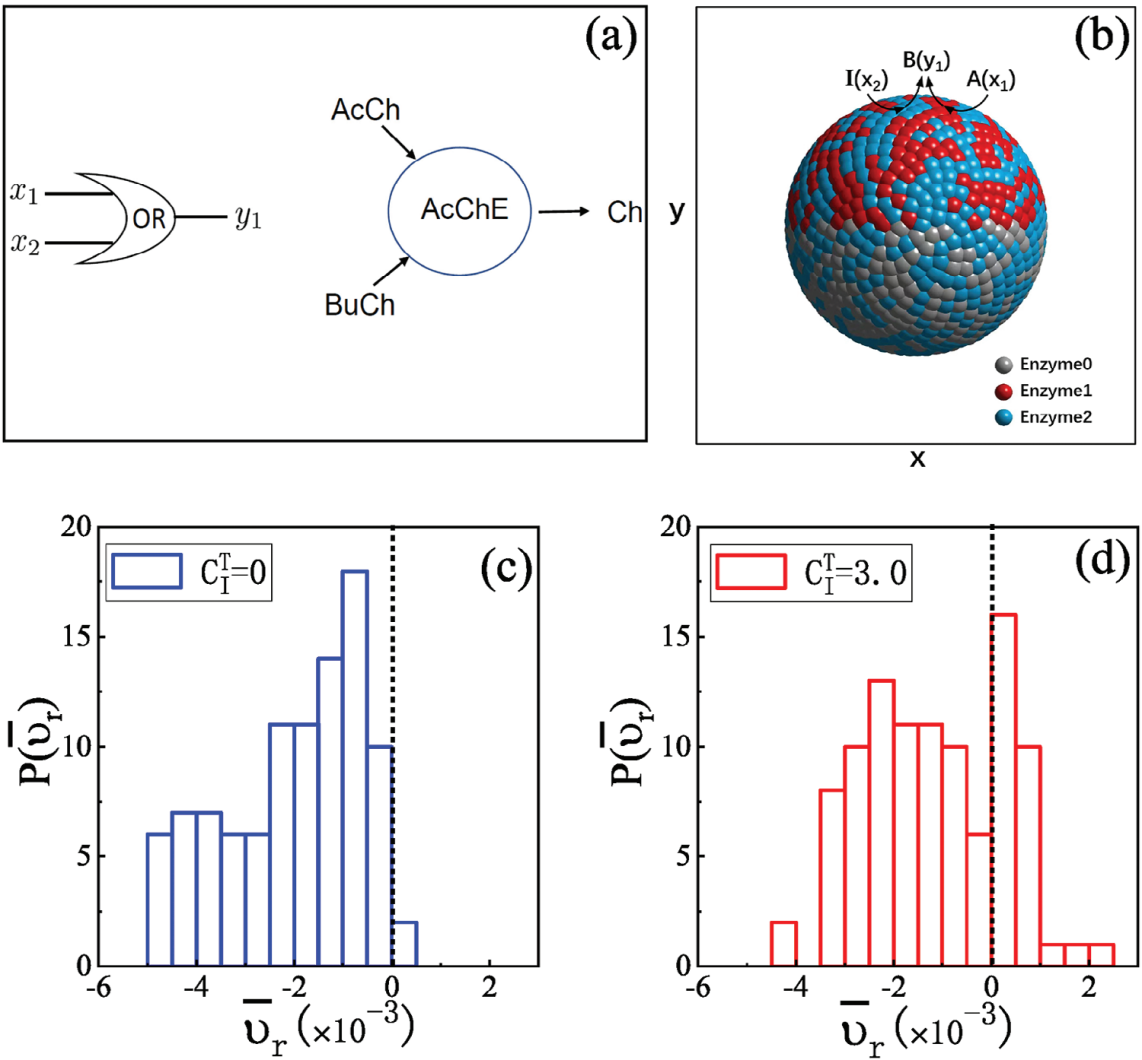
a) An enzymatic reaction that simulates an OR gate. The enzyme acetylcholinesterase (AcCHE) catalyzes the decomposition of both acetylcholine (AcCH) and butyrycholine (BuCh) to the product choline (Ch). The inputs to the OR gate are *x*_1_ = AcCh and *x*_2_ = BuCh and the output is *y*_1_ = Ch. b) Janus colloid with equal numbers, $N_{E1}^{u}=N_{E2}^{u}=250$, of E_1_ and E_2_ enzymes randomly distributed on the upper *H*_*u*_ hemisphere. Likewise, equal numbers, $N_{E1}^{\ell }=N_{E0}^{\ell }=250$, of E_1_ and E_0_ enzymes are randomly distributed on the lower hemisphere H_ℓ_. Both substrates A and I can be converted to B by E_1_ and E_2_, respectively. Enzymes E_0_ are chemically inactive. The interaction parameters are chosen to be ϵ_A_ = ϵ_I_ = 2.0 and ϵ_B_ = 0.1. The distribution of average radial velocity ${\bar{\upsilon }}_{r}=\left(R_{\text{CS}}\left(t_{\text{traj}}\right)-R_{\text{CS}}^{0}\right)/t_{\text{traj}}$ of colloids for OR gate thresholds c) $C_{I}^{T}=0$ and d) $C_{I}^{T}=3.0$, where *t*_traj_ is the corresponding time for the actual trajectory *R*_CS_(*t*_traj_) = ∣**r**_c_ − **r**_s_∣ traveled by the colloid within the simulation time, and initial $R_{\text{CS}}^{0}$ is 15.0. ${\bar{\upsilon }}_{r}$ is depicted in a coordinate system with the initial position of the colloid as the origin and the positive direction pointing outward along the radial axis (from the source to the colloid). The simulation stops when the colloid touches the source or outer boundary (a circle with a radius of 40.0 centered around the source). In (c) and (d), the system size is 80 × 80 × 14, the simulation time is 3 ×  10^5^, and the data is plotted from 100 independent realizations.

An OR gate is shown in **Figure** **4**a. This figure also shows an enzymatic implementation of such a gate that has been discussed in the literature.^[^ ^55^ ^]^ The single enzyme acetylcholinesterase (AcCHE) can catalyze the decomposition of both acetylcholine (AcCH) and butyrycholine (BuCh) to give the common product choline (Ch), so that it mimics the action of an OR gate.

The task using the OR gate is a variation on that for the INH gate above but shows how a gate can be used to change the propulsion direction of a Janus colloid through a chemotactic effect in order to be captured by a source. We again have a source sphere that produces I by consuming A. However, the colloid is Janus particle where one hemisphere *H*_*u*_ is randomly covered by enzymes E_1_ and E_2_ with equal probability 1/2, while *H*_ℓ_ is randomly covered by E_1_ and inactive E_0_ enzymes, also with probability 1/2, as shown in Figure 4b. The reaction on E_1_ is $A\underset{E_{1}}{\overset{\kappa_{1}}{\to }}B$ and that on E_2_ is $I\underset{E_{2}}{\overset{\kappa_{2}}{\to }}B$. In our example the Janus colloid supports two different enzymatic reactions and functions as an OR gate since the common product B is produced if either A or I are inputs to the colloid.

Initially, the fluid has only A solute particles distributed uniformly. The potential parameters satisfy ϵ_A_ = ϵ_I_ > ϵ_B_. The Janus colloid uses the reaction on *E*_1_ ($A\underset{E_{1}}{\overset{\kappa_{1}}{\to }}B$) for propulsion and, since ϵ_A_ > ϵ_B_, the propulsion force is directed along $\hat{u}$ pointing toward the *H*_*u*_ cap. On time scales longer than the colloid orientational relaxation time, the active Janus colloid will undergo diffusive motion with an enhanced effective diffusion coefficient. If the reaction A → I does not take place so that the source sphere S is inactive, with a low probability the Janus colloid may encounter S during its random walk. If the source converts A → I producing an inhomogeneous concentration field in its vicinity, but the colloid reaction $I\underset{E_{2}}{\overset{\kappa_{2}}{\to }}B$ is not activated, that is, only $A\underset{E_{1}}{\overset{\kappa_{1}}{\to }}B$ occurs on the colloid, the colloid still displays enhanced effective diffusion since ϵ_A_ = ϵ_I_.

If the source is active and the colloid senses the I concentration field, then the reaction $I\underset{E_{2}}{\overset{\kappa_{2}}{\to }}B$ takes place on the E_2_ sites that are uniformly distributed on the colloid (see Figure 4b) simulating an OR gate. Now B will also be preferentially produced on the face of the colloid that points toward the source, since species I has a higher concentration near to the source (see Equation (5)). In general, this face will not point in the same direction as vector $\hat{u}$. As a result the colloid diffusiophoretic force lies in a direction determined by the combined production of B due to both catalytic reactions. This biases the propulsion direction toward the source, and leads to capture of the colloid.

We suppose that the OR gate operates when the local concentration of I (*c*_I_) around surface enzyme beads exceeds a pre‐set threshold value $c_{I}^{T}$. Two examples, $c_{I}<c_{I}^{T}$ and $c_{I}>c_{I}^{T}$, are shown in Figure 4c,d. For $c_{I}<c_{I}^{T}$, the OR gate is not operative initially. Only when the colloid randomly moves into the region where $c_{I}>c_{I}^{T}$, will the enzymes be activated and the OR gate perform its task. Compared to the case with $c_{I}>c_{I}^{T}$, the capture probability is lower and consequently the capture time τ_C_ is larger: From $c_{I}^{T}=0$ in panel (c) to $c_{I}^{T}=3.0$ in panel (d), the average capture time ${\bar{\tau }}_{C}$ has roughly doubled, and the capture probabilities decrease from 0.98 to 0.71 during the simulation time. The average radial velocity ${\bar{\upsilon }}_{r}$ of each trajectory in the ensemble can be measured, and its probability distribution, shown in Figure 4c,d, can effectively characterize the dynamical behavior caused by different threshold values of the OR gate. For $c_{I}^{T}=0$, most of the distribution lies in the ${\bar{\upsilon }}_{r}<0$ region, implying that the colloid exhibits chemotactic behavior. For $c_{I}^{T}=3.0$, a portion of the distribution has ${\bar{\upsilon }}_{r}>0$, so that the colloids in those realizations move away from the source. The distribution in ${\bar{\upsilon }}_{r}>0$ increases as $c_{I}^{T}$, until it reaches a maximum value when $c_{I}^{T}=c_{0}$.

## 3 Conclusion

The results in this paper showed how small enzyme networks on colloidal motors could be used to construct AND, OR, and INH chemical logic gates, and how these gates can enable the active colloids to perform simple tasks. Other logic gates can be constructed in a similar fashion. For example, concatenated OR, AND, and XOR gates have been constructed using four coupled enzyme reactions,^[^ ^69^ ^]^ while the NAND gate can be constructed from AND gate followed by a NOT gate. The simulation results and continuum models provide detailed information on the way surface enzyme networks function to produce the inhomogeneous concentration fields that play an important part in how chemical gates function, how fuel is consumed in the network, and how the colloid is propelled.

The examples of sensing tasks presented above, while simple, show how an active colloid with chemical gates can use propulsion and sensing to change its dynamics to achieve a goal. Small enzyme networks of the sort shown in these examples can be used to construct all the logic gates, and strategies for constructing such gates have been described recently.^[^ ^56^ ^]^ The results lay the foundation for further research. The experiments on micromotors with a gated pH responsive DNA nanoswitch mentioned earlier are laboratory examples of how such gated active colloids could be used in applications.^[^ ^42^, ^43^ ^]^

There have been other studies that consider how active motion can be combined with logic functions or rules to yield complex dynamics; for example, model gates have been used in the context of active microfluidics,^[^ ^70^ ^]^ run‐and‐tumble particles with simple rules for gates,^[^ ^71^ ^]^ and the use of chemical signals to communicate among self‐propelled particles.^[^ ^72^ ^]^ In the work presented here, model protein networks were used to build the chemical gates, and the simulations of gate operations were carried out at a coarse‐grained microscopic level that takes into account the full reactive dynamics and fluid flows that underlie the propulsion mechanism and gate operations of the colloids, along with their interactions with their environments.

Proteins generally have a high specificity for substrate molecules enabling them to sense their temporally and spatially varying environments. Thus, the proteins on the surfaces of the colloids, either in chemical gates or acting singly, can be used to enable diverse sensing capability. The work presented here could be extended to treat these more complex and interesting situations, and may serve to interpret or suggest future experimental work on active colloids that sense and autonomously respond to their environments. Such applications can involve logical circuits built from these gates^[^ ^51^, ^55^ ^]^ that arise from the self‐assembly of collections of colloids. Such assemblies could be designed to carry out specific computations needed to accomplish a task, or evolve to yield assemblies with unexpected computational ability. More generally, the collective behavior of such logical active elements, with their rich communication with the environment and with each other, may lead to the emergence of novel dynamic phases.^[^ ^73^, ^74^ ^]^

## 4 Experimental Section

### Continuum Model

These calculations gave some details that entered Equations (3) and (4). The enzymes with surface reactions in Equations (1) and (2) have fractions $f_{1}^{u}=f_{1}^{\ell }=1/2$ and $f_{2}^{u}=1/2$ and $f_{2}^{\ell }=0$. Since the total global concentration is conserved, *c*_A_ + *c*_B_ + *c*_I_ = *c* = *c*_0_ = const, it is convenient to consider the variables c(r,t), $c_{B}\left(r,t\right)$ and $c_{I}\left(r,t\right)$ which satisfy the uncoupled equations

(6) $$\begin{array}{ccc} \partial_{t}c\left(r,t\right) & = & D\nabla^{2}c \end{array}$$

(7) $$\begin{array}{ccc} \partial_{t}c_{B}\left(r,t\right) & = & D\nabla^{2}c_{B}-k_{b}^{B}c_{B} \end{array}$$

(8) $$\begin{array}{ccc} \partial_{t}c_{I}\left(r,t\right) & = & D\nabla^{2}c_{I}-k_{b}^{I}c_{I} \end{array}$$

where *k*_D_ = 4π*DR*. These fields were subjected to the following boundary conditions on the surface of the colloid:

(9) $$\begin{array}{ccc} k_{D}R\partial_{r}c\left(r,\theta ,t\right){|}_{R} & = & 0 \end{array}$$

(10) $$\begin{array}{ccc} k_{D}R\partial_{r}c_{B}\left(r,\theta ,t\right){|}_{R} & = & -k_{2}\Theta_{u}c_{I}\left(R,\theta ,t\right) \end{array}$$

(11) $$\begin{array}{ccc} k_{D}R\partial_{r}c_{I}\left(r,\theta ,t\right){|}_{R} & = & \left(k_{2}\Theta_{u}+k_{1}\right)c_{I}\left(R,\theta ,t\right)\\ & & +k_{1}c_{B}\left(R,\theta ,t\right)-k_{1}c_{0} \end{array}$$

where Θ_*u*_ is a Heaviside function that is unity on the upper hemisphere and zero otherwise, while at infinity we have lim_*r* → ∞_ *c*(*r*, θ, *t*) = *c*_0_, lim_*r* → ∞_ *c*_*B*_(*r*, θ, *t*) = 0 and lim_*r* → ∞_ *c*_I_(*r*, θ, *t*) = 0.

Henceforth, the steady state versions of these equations were considered. The steady state solutions of the fluid phase reaction‐diffusion can be written as

(12) $$\begin{array}{ccc} c(r,\theta ) & = & c_{0}+\sum_{\ell =0}^{\infty }a_{\ell }^{c}{\left(\frac{R}{r}\right)}^{\ell +1}P_{\ell }\left(\cos\theta \right) \end{array}$$

(13) $$\begin{array}{ccc} c_{B}\left(r,\theta \right) & = & \sum_{\ell =0}^{\infty }a_{\ell }^{B}f_{\ell }^{B}\left(r\right)P_{\ell }\left(\cos\theta \right) \end{array}$$

(14) $$\begin{array}{ccc} c_{I}\left(r,\theta \right) & = & \sum_{\ell =0}^{\infty }a_{\ell }^{I}f_{\ell }^{I}\left(r\right)P_{\ell }\left(\cos\theta \right) \end{array}$$

where, for α = B, I,

(15) $$f_{\ell }^{\alpha }\left(r\right)=\frac{\sqrt{\nu_{b}^{\alpha }R}\;K_{\ell +\frac{1}{2}}\left(\nu_{b}^{\alpha }r\right)}{\sqrt{\nu_{b}^{\alpha }r}\;K_{\ell +\frac{1}{2}}\left(\nu_{b}^{\alpha }R\right)}$$

with $\nu_{b}^{\alpha }=\sqrt{k_{b}^{\alpha }/D}$ and *K*_*n*_(*x*) an associated Bessel function of the second kind. The boundary condition for *c* is

(16) $$\partial_{r}c\left(r,\theta \right){|}_{R}=-k_{D}R\sum_{\ell =0}^{\infty }a_{\ell }^{c}\frac{\left(\ell +1\right)}{R}P_{\ell }\left(\cos\theta \right)=0$$

from which it was concluded that $a_{\ell }^{c}=0$ for all ℓ. Therefore *c*(*r*, θ) = *c*_0_ as expected. The boundary condition for *c*_B_ yields

(17) $$\frac{2Q_{\ell }^{B}}{2\ell +1}a_{\ell }^{B}=\frac{k_{2}}{k_{D}}\sum_{m=0}^{\infty }N_{\ell m}a_{m}^{I}$$

where *x* = cos θ, $Q_{\ell }^{\alpha }$ for α = B, I is defined by,

(18) $$Q_{\ell }^{\alpha }=\frac{\left(\nu_{b}^{\alpha }R\right)\;K_{\ell +\frac{3}{2}}\left(\nu_{b}^{\alpha }R\right)}{K_{\ell +\frac{1}{2}}\left(\nu_{b}^{\alpha }R\right)}-\ell$$

and the matrix element $N_{\ell m}=\int_{0}^{1}dx\;P_{\ell }\left(x\right)P_{m}\left(x\right)$ could be computed analytically. After some rearrangement of the *c*_B_ and *c*_I_ boundary conditions a closed linear equation could be obtained for the $a_{\ell }^{I}$ coefficients:

(19) $$a_{\ell }^{I}=2\frac{k_{1}}{k_{D}}c_{0}{\left(M^{-1}\right)}_{\ell 0}$$

where the elements of M are

(20) $$M_{\ell m}=\frac{2}{2\ell +1}\left(Q_{\ell }^{I}+\frac{k_{1}}{k_{D}}\right)\delta_{\ell m}+\frac{k_{2}}{k_{D}}\left(1+\frac{k_{1}}{k_{D}}\frac{1}{Q_{\ell }^{B}}\right)N_{\ell m}$$

Once these $a_{\ell }^{I}$ coefficients were obtained, the coefficients $a_{\ell }^{B}$ can be found after substitution into Equation (17); thus, the concentration fields *c*_I_(*r*, θ), *c*_I_(*r*, θ) and *c*_A_(*r*, θ) can be obtained. The upper limit on the sums over Legendre polynomials was sufficiently large (ℓ ≈ *XX*) to insure convergence of the series to the desired accuracy.

Next, these results would be used to compute the propulsion velocity of the colloid. The expression for the diffusiophoretic velocity is given by^[^ ^32^, ^75^ ^]^

(21) $$V_{u}=V_{u}\hat{u}=\frac{1}{1+2b/R}\sum_{\alpha }b_{\alpha }{\bar{\nabla_{s}c_{\alpha }\left(r\right)}}^{S}$$

where α = A, I, B and, as noted earlier, the overline denotes an average over the surface of the colloid, ${\bar{O}}^{S}=\frac{1}{4\pi R^{2}}\int dr\;\delta \left(r-R\right)O$, and $b_{\alpha }=\frac{k_{B}T}{\eta }\left(K_{\alpha }^{\left(1\right)}+bK_{\alpha }^{\left(0\right)}\right)$ with *b* the slip length and

(22) $$K_{\alpha }^{\left(n\right)}=\int_{R}^{R+r_{c}}dr\;{\left(r-R\right)}^{n}\left(e^{-\beta V_{\alpha c}}-1\right)$$

and *r*_c_ the distance beyond which the solute‐colloid interactions vanish.

For this system (using *c*_A_ = *c*_0_ − *c*_I_ − *c*_B_)

(23) $$\begin{array}{ccc} V_{u} & = & \frac{1}{1+2b/R}\frac{k_{B}T}{\eta }\left[\Lambda_{IA}{\bar{\hat{u}\cdot \nabla_{s}c_{I}\left(R,\theta \right)}}^{S}\right.\\ & & \left.\;+\Lambda_{\text{BA}}{\bar{\hat{u}\cdot \nabla_{s}c_{B}\left(R,\theta \right)}}^{S}\right] \end{array}$$

where $\Lambda_{\alpha \alpha^{\prime }}=\Lambda_{\alpha \alpha^{\prime }}^{\left(1\right)}+b\Lambda_{\alpha \alpha^{\prime }}^{\left(0\right)}$ with

(24) $$\Lambda_{\alpha \alpha^{\prime }}^{\left(n\right)}=\int_{R}^{R+r_{c}}dr\;{\left(r-R\right)}^{n}\left(e^{-\beta V_{\alpha c}}-e^{-\beta V_{\alpha^{\prime }c}}\right)$$

This is Equation (3) in the text with *b* = 0 for velocity stick boundary conditions. Since $\hat{u}\cdot \nabla_{s}=-\frac{\sin\theta }{r}\partial_{\theta }$. This equation can also be written as

(25) $$\begin{array}{ccc} V_{u} & = & -\frac{1}{1+2b/R}\frac{k_{B}T}{R\eta }\left[\Lambda_{IA}\;{\bar{\sin\theta \;\partial_{\theta }c_{I}\left(R,\theta \right)}}^{S}\right.\\ & & \left.+\Lambda_{\text{BA}}\;{\bar{\sin\theta \;\partial_{\theta }c_{B}\left(R,\theta \right)}}^{S}\right] \end{array}$$

Integrating by parts in the surface averages and substituting the expression for *c*_I_(*R*, θ) and *c*_B_(*R*, θ) the final result for the colloid velocity was obtained,

(26) $$\begin{array}{ccc} V_{u} & = & -\frac{2}{1+2b/R}\frac{k_{B}T}{R\eta }\frac{k_{1}}{k_{D}}c_{0}\\ & & \times \sum_{m=0}^{\infty }\left[\frac{2}{3}\Lambda_{IA}\;\delta_{m1}+\frac{k_{2}}{k_{D}}\frac{1}{Q_{1}^{B}}\Lambda_{BA}\;N_{1m}\right]{\left(M^{-1}\right)}_{m0} \end{array}$$

### Direct Supply of Species *I* from Fluid Phase

The above results could be compared with a system where fuel I was supplied in the bulk:

(27) $$\begin{array}{ccc} I\overset{\kappa_{2}}{\to }B, & \; & \mathrm{on}\;\mathrm{hemispherical}\;\mathrm{surface} \end{array}$$

(28) $$\begin{array}{ccc} B\overset{k_{b}^{B}}{\to }I, & \; & \mathrm{in}\;\mathrm{the}\;\mathrm{fluid}\;\mathrm{phase} \end{array}$$

The reaction‐diffusion equations are

(29) $$\begin{array}{ccc} \partial_{t}c_{B}\left(r,t\right) & = & D\nabla^{2}c_{B}-k_{b}^{B}c_{B} \end{array}$$

(30) $$\begin{array}{ccc} \partial_{t}c_{I}\left(r,t\right) & = & D\nabla^{2}c_{I}+k_{b}^{B}c_{B} \end{array}$$

with boundary conditions

(31) $$\begin{array}{ccc} k_{D}R\partial_{r}c_{B}\left(r,\theta ,t\right){|}_{R} & = & -k_{2}\Theta_{u}c_{I}\left(R,\theta ,t\right) \end{array}$$

(32) $$\begin{array}{ccc} k_{D}R\partial_{r}c_{I}\left(r,\theta ,t\right){|}_{R} & = & k_{2}\Theta_{u}c_{I}\left(R,\theta ,t\right) \end{array}$$

while at infinity we have lim_*r* → ∞_ *c*_B_(*r*, θ, *t*) = 0 and lim_*r* → ∞_ *c*_I_(*r*, θ, *t*) = *c*_0_.

Similar to the calculation given above, the solutions to the reaction‐diffusion equations can be written as

(33) $$c_{B}\left(r,\theta \right)=\sum_{\ell =0}^{\infty }{\tilde{a}}_{\ell }^{B}f_{\ell }^{B}\left(r\right)P_{\ell }\left(\cos\theta \right)$$

with *c*_I_(*r*, θ) = *c*_0_ − *c*_B_(*r*, θ) that follows from number conservation. Determining the coefficients from the boundary conditions gives

(34) $${\tilde{a}}_{\ell }^{B}=\sum_{m=0}^{\infty }{\left({\tilde{M}}^{-1}\right)}_{\ell m}{\tilde{E}}_{m}$$

where ${\tilde{E}}_{m}=\left(k_{2}/k_{D}\right)c_{0}\int_{0}^{1}dxP_{m}\left(x\right)$ and

(35) $${\tilde{M}}_{\ell m}=\frac{2Q_{\ell }^{B}}{2\ell +1}\delta_{\ell m}+\frac{k_{2}}{k_{D}}N_{\ell m}$$

Using this result the motor velocity is

(36) $$V_{u}=\frac{1}{1+2b/R}\frac{k_{B}T}{R\eta }\frac{2}{3}\Lambda_{\text{IB}}{\tilde{a}}_{1}^{B}$$

### Simulation Model and Parameters

The active colloid with radius *R* = 4.0 and mass *M*_c_ was constructed from spherical beads linked by stiff harmonic bonds to ensure that the spherical shape of the colloid was maintained during the evolution of the system. The *N*_E_  =  1000 surface beads with radius σ = 1.0 were coarse‐grained enzymatic sites. The colloid was contained in a cubic simulation box with size *V* = *L*_*x*_ × *L*_*y*_ × *L*_*z*_ containing reactive particles. Periodic boundary conditions were applied.

The point‐like substrate molecules with mass *m* interacted with the surface enzyme beads through repulsive Lennard–Jones (LJ) potentials, *V*_LJ_(*r*) = 4ϵ_α_((σ/*r*)^12^ − (σ/*r*)^6^ + 1/4)Θ(*r*_c_ − *r*), where Θ is a Heaviside function, α labels species, and *r*_*c*_ = 3 + 2^1/6^ = 4.125 is the cutoff distance beyond which the potential is zero.

For simulation where the colloid was confined to the center plane in a simulation box with a slab geometry, five beads were selected: one at the center‐of‐mass of the colloid, and others on a circle with *r* = 3.5 in the *x*–*y* plane at *z* = *L*_*z*_/2. They interacted with the walls at *z* = 0 and *z* = *L*_*z*_ through a 9‐3 LJ potential, $V_{\text{LJ}}^{93}\left(r\right)=\varepsilon_{w}\left[{\left(\sigma_{w}/r\right)}^{9}-{\left(\sigma_{w}/r\right)}^{3}\right]$, where ϵ_w_ and σ_w_ are the wall energy and distance parameters, respectively. The interaction between the fluid particles and the source sphere as 6–12 repulsive LJ potentials with ϵ = 0.1.

Reactive events that converted substrate to product took place on the enzyme beads *E*_ν_ (ν labels different enzymes) with probabilities $p_{\nu }^{R}$.^[^ ^76^ ^]^ For the continuum calculation, the reaction rate constant for the colloid could be estimated from the kinetic theory expression,

(37) $$k_{\nu }=p_{\nu }^{R}r_{c}^{2}\sqrt{8\pi k_{B}T/\mu_{\text{cs}}}$$

and the reduced mass is µ_cs_ = (*M*_c_ *m*)/(*M*_c_ + *m*).

The system was evolved using hybrid molecular dynamics‐multiparticle collision (MPC) dynamics.^[^ ^65^, ^66^, ^67^, ^68^ ^]^ Simulation results were reported in dimensionless units based on energy ϵ, mass *m*, and cell length *a*_0_. The time was in units of $t{\left(ma_{0}^{2}/\varepsilon \right)}^{1/2}\to t$, distance parameter *r*/*a*_0_ → *r*, and temperature *k*_B_ *T*/ϵ → *T*. The system temperature *T* was $\frac{1}{6}$. The average number of fluid particles per cell was *c*_0_ = 10.2. The mass of the colloid was given by $M_{c}=\frac{4}{3}\pi R^{3}c_{0}$, so that the colloid was approximately neutrally buoyant. The MPC rotation angle was $\phi =\frac{\pi }{2}$ and collision time interval was τ_MPC_ = 0.5. The velocity Verlet algorithm was used to integrate the Newton's equation of motion with τ_MD_ = 0.01. The Schmidt number was *S*_c_ = 1.39. Other parameters: harmonic spring force constant *k*_s_ = 60, wall ϵ_w_ = 5.0, σ_w_ = *L*_*z*_/2, fluid rate constants $k_{b}^{B}=k_{b}^{I}=0.001$, common diffusion coefficient D = 0.097, viscosity from MPC expression η = 1.35. The values of ϵ_α_ are given in the text. Grid shifting was employed to ensure Galilean invariance.^[^ ^77^ ^]^

## Conflict of Interest

The authors declare no conflict of interest.

## Data Availability

The data that support the findings of this study are available from the corresponding author upon reasonable request.
